# Identification and characterization of microRNA in the lung tissue of pigs with different susceptibilities to PCV2 infection

**DOI:** 10.1186/s13567-018-0512-3

**Published:** 2018-02-15

**Authors:** Ping Zhang, Liyuan Wang, Yanping Li, Ping Jiang, Yanchao Wang, Pengfei Wang, Li Kang, Yuding Wang, Yi Sun, Yunliang Jiang

**Affiliations:** 1 Shandong Provincial Key Laboratory of Animal Biotechnology and Disease Control and Prevention, Shandong Agricultural University, 61 Daizong Street, Taian, 271018 Shandong China; 2 College of Veterinary Medicine, Nanjing Agricultural University, Nanjing, 210095 China

## Abstract

Porcine circovirus type 2 (PCV2) is the primary cause of post-weaning multisystemic wasting syndrome (PMWS) and other PCV-associated diseases. According to our previous RNA-sequencing analysis, the differences in the susceptibility to PCV2 infection depended on the genetic differences between the Laiwu (LW) and Yorkshire × Landrace crossbred (YL) pigs, but the cellular microRNA (miRNA) that are differentially expressed between the LW and YL pigs before and after PCV2 infection remain to be determined. In this study, high-throughput sequencing was performed to determine the abundance and differential expression of miRNA in lung tissues from PCV2-infected and PCV2-uninfected LW and YL pigs. In total, 295 known and 95 novel miRNA were identified, and 23 known and 25 novel miRNA were significantly differentially expressed in the PCV2-infected vs. PCV2-uninfected LW pigs and/or the PCV2-infected vs. PCV2-uninfected YL pigs. The expression levels of *ssc*-*miR*-*122*, *ssc*-*miR*-*192*, *ssc*-*miR*-*451*, *ssc*-*miR*-*486*, and *ssc*-*miR*-*504* were confirmed by quantitative real-time PCR (qRT-PCR). Analysis of the potential targets of the four up-regulated miRNA (i.e., *ssc*-*miR*-*122*, *ssc*-*miR*-*192*, *ssc*-*miR*-*451* and *ssc*-*miR*-*486*) identified pathways and genes that may be important for disease resistance. Among the up-regulated miRNA, *ssc*-*miR*-*122* can repress the protein expression and viral DNA replication of PCV2 and down-regulate the expression of the *nuclear factor of activated T*-*cells 5* (*NFAT5*) and *aminopeptidase puromycin sensitive* (*NPEPPS*) by binding to their 3′ untranslated region (3′UTR) in PK15 cells. Therefore, *ssc*-*miR*-*122* may indirectly suppress PCV2 infection by targeting genes related to the host immune system, such as *NFAT5* and *NPEPPS*.

**Electronic supplementary material:**

The online version of this article (10.1186/s13567-018-0512-3) contains supplementary material, which is available to authorized users.

## Introduction

Porcine circovirus (PCV) was first considered a contaminant in a porcine kidney cell line in 1974 and was described in greater detail in 1982. In the late 1990s, PCV2 was found to be associated with post-weaning multisystemic wasting syndrome (PMWS), which is currently considered one of the most important swine diseases worldwide [1]. PCV2 belongs to the *Circoviridae* family, which is characterized by a genome consisting of single-strand circular DNA with 1768-9 nucleotides (nt) [2, 3]. PCV2-affected pigs show wasting and progressive weight loss, enlarged lymph nodes, and respiratory distress, jaundice, and occasional diarrhea [4]. Morbidity in PCV2-affected farms is commonly 4–30% (occasionally as high as 50–60%), and mortality ranges from 4 to 20% [5]. Because of the increased mortality rates and the impact on weight gain, PCV2 has had a serious economic impact on the swine production industry [6]. The replication patterns of PCV2 in pulmonary alveolar macrophages are different among macrophages derived from different conventional crossbred pigs [7].

MicroRNA (miRNA) are ~21–23 nt small RNA (sRNA) molecules that regulate gene expression at the post-transcriptional level [8]. Currently, miRNA have been estimated to constitute 1–5% of animal genes and collectively regulate up to 30% of genes; therefore, miRNA are among the most abundant regulators [9]. No miRNA encoded by PCV2 genomic DNA were detected in tonsil and mediastinal lymph node tissues from PCV2-infected pigs [10]. In PK15 cells expressing PCV2 ORF1, ORF2 and ORF3, 51, 74 and 32 miRNA were identified, respectively, differing in abundance from those in the controls [11]. In PCV2-infected Landrace × Yorkshire pigs, *miR*-*126*-*3p*, *126*-*5p*, *129a*, *let*-*7d*-*3p* and *let*-*7b*-*3p* were up-regulated, while *miR*-*193a*-*5p*, *574*-*5p* and *34a* were down-regulated in the mediastinal lymph node [12]. These differentially expressed miRNA are mainly involved in the regulation of the immune system and cell proliferation. Although certain PCV2 infection-associated miRNA have been identified, pig-breed-specific miRNA that are differentially expressed and likely account for the resistance to PCV2 infection have not been characterized.

The Laiwu (LW) pig is a Chinese indigenous pig breed from Shandong Province that is well-known for its extremely high intramuscular fat content of > 10%. The LW pig also exhibits a higher resistance to certain infectious diseases, including PCV2. In our previous study, PCV2-infected Yorkshire × Landrace (YL) pigs exhibited serious clinical features that are typical of PCV2 disease, particularly severe lesions in the lungs, such as congestion, bleeding, interstitial pneumonia and lymphocyte infiltration, while the PCV2-infected LW pigs showed only a few clinical symptoms; at 35 days post-infection (dpi), the PCV2 DNA copy in the YL pigs was significantly higher than that in the LW pigs [13]. In this study, using Illumina/Solexa high-throughput sequencing, we identified the differentially expressed miRNA in the lung tissues between LW and YL pigs prior to and post PCV2 infection and further characterized the role of *miR*-*122* in conferring higher resistance to PCV2 infection.

## Materials and methods

### Sample collection

Fifteen purebred LW and 15 YL weaned pigs that were validated to be free of PCV2, porcine circovirus type 1 (PCV1), porcine reproductive and respiratory syndrome virus (PRRSV) and porcine parvovirus (PPV) were raised under identical conditions on the same farm. All pigs were randomly divided into the following four groups: PCV2-infected LW pigs (LW-i, *n* = 10), PCV2-uninfected LW pigs (LW-u, *n* = 5), PCV2-infected YL pigs (YL-i, *n* = 10) and PCV2-uninfected YL pigs (YL-u, *n* = 5). The procedures used for the pig management and PCV2 inoculation have been previously described [13, 14]. Briefly, the PCV2 strain (named PCV2-SD) used in this experiment was isolated from suspected PMWS pigs in the Shandong province. The virus genome of PCV2-SD is 1767 bp in size and phylogenetic analysis based on both the whole genome and ORF2 sequences indicate that the genotype of PCV2-SD is PCV2b. Each pig from LW-i and YL-i groups was intramuscularly injected with 3 mL PCV2-SD solution with 10^3.8^TCID_50_ (50% tissue culture infective dose)/mL. The pigs from LW-u and YL-u groups were treated similarly with an identical volume of phosphate buffered saline (PBS). During the experimental period, clinical signs were monitored daily and the copy number of PCV2 DNA in serum was detected by quantitative real-time PCR (qRT-PCR) at 0, 4, 7, 10, 14, 21, 28 and 35 dpi. From 7 dpi, PCV2 virus could be detected from the serums of PCV2-infected pigs, indicating that the intramuscular injection was successful. The mean copy number of PCV2 DNA in serum significantly increased in both PCV2-infected LW and YL pigs at 14 dpi. PCV2-infected YL pigs exhibited serious clinical signs typifying PMWS, while the PCV2-infected LW pigs showed slightly clinical symptoms. The lung tissue of PCV2-infected YL pigs showed serious lesions, while they were not observed in PCV2-infected LW pigs [13]. All pigs were sacrificed at 35 dpi and tissue samples were frozen in liquid nitrogen or preserved by immersion in 10% neutral-buffered formalin.

### sRNA library preparation and deep-sequencing

Four sRNA libraries of LW-u, LW-i, YL-u and YL-i were constructed using the homogenized and pooled total RNA from four individuals selected from each group as previously described [15–17]. The construction of pooled small RNA libraries is certainly better than pooled total RNA before Illumina sequencing. To minimize deviation of this procedure compared with the results coming from each individual per group, more miRNA were validated by qRT-PCR in this study. Total RNA was extracted from the lung tissues of PCV2-infected and uninfected pigs of each breed with TRIzol^®^ Reagent (Invitrogen Life Technologies, Carlsbad, CA, USA). Briefly, for each sample, 20 μg of total RNA and a Small RNA Sample Prep Kit (Illumina, San Diego, CA, USA) were used for library construction according to the manufacturer’s instructions. Then, fractions between 18 and 30 nt were removed and purified using 15% Tris–borate-EDTA denaturing polyacrylamide gel electrophoresis (PAGE). Subsequently, the 3′ and 5′ RNA adaptors were ligated to the purified fragments with T4 RNA ligase in proper order. The cDNA from the adaptor-ligated sRNA were then amplified by RT-PCR with 15 cycles. The products (90-bp sRNA + adaptors) after purification on 4% agarose gels were used for sequencing on an Illumina 1G Genome Analyzer at Beijing Genomics Institute (BGI, Shenzhen, China). After masking and removing the redundant reads, the clean reads were processed for further analysis.

### Alignment, annotation, and clustering of reads

The initial output was converted into raw sequence data in a base-calling step. The reads were sorted according to the barcode index, and the adapter sequences were trimmed. The remaining 18–30 nt identical high-quality sequences were counted, and the unique sequences and their associated read counts were mapped to the *Sus scrofa* genome assembly with no mismatches using SOAP v.1.11 (short oligonucleotide alignment program) [18] to analyze their expression and distribution. The sequences that perfectly matched the reference genome sequence were retained for further analysis. The sequences were aligned against both known miRNA precursors and mature miRNA deposited in miRBase [19] (Release 21) to identify the conserved miRNA. The clean reads were compared with the sRNA deposited in the GenBank and Rfam [20] databases. The sRNA sequences were annotated using the tag2 annotation software developed by BGI. Because some sRNA tags may be mapped to more than one sRNA category, the following priority rule was used to ensure uniqueness: sRNA (GenBank > Rfam) > known miRNA > repeat > exon > intron [21]. The characteristic hairpin structures of the miRNA precursor sequences were used to predict the novel miRNA.

### Differential expression analysis and hierarchical clustering of miRNA

To compare the miRNA expression levels between any two samples, the expression of the miRNA in the two series (LW-i vs. LW-u and YL-i vs. YL-u) were normalized to obtain the expression of transcripts per million reads. If the normalized expression of a given miRNA was zero, its expression value was set to 0.01. If the normalized expression of a given miRNA was less than 1 in both samples of a sample pair, the miRNA was removed from the differential expression analysis. The fold-change and *P* value of each miRNA in each sample pair were calculated using previously published criteria [15–17]. A hierarchical clustering analysis of the miRNA expression levels was performed using PermutMatrix software. The relative expression level of each miRNA was calculated as the total number of reads in the four libraries.

### qRT-PCR

Differentially expressed miRNA and target mRNA were validated using qRT-PCR according to the manufacturer’s protocol. Lung tissues from 16 pigs (four groups with *n* = 4 per group) sequenced by BGI were used as substrates for the qRT-PCR, which was performed using an Mx3000p^TM^ SYBR Green qRT-PCR Analyzer (Stratagene, CA, USA).

For the miRNA detection, the SYBR^®^PrimeScript™ miRNA RT-PCR Kit (TaKaRa Biotechnology Co., Ltd., Japan) was used. The reverse transcription system included 10 μL of 2 × miRNA reaction buffer, 2 μL of 0.1% BSA, 2 μL of miRNA PrimeScript^TM^ RT Enzyme Mix, 2 μL of total RNA (1 mg/mL), and up to 20 μL RNase-free H_2_O. The reverse transcription program was as follows: 37 °C for 60 min, followed by 85 °C for 5 s. The cDNA was then used for the real-time PCR quantification of the miRNA using a miRNA-specific primer and the Uni-miR qPCR primer developed by TaKaRa. 5S rRNA was used as an endogenous control as listed in Table 1. The real-time PCR reaction mixture was prepared on ice and comprised 10 μL of 2 × SYBR^®^ Premix Ex Taq™ II, 0.8 μL of PCR forward primer (10 μM), 0.8 μL of Uni-miR qPCR primer (10 μM), 0.4 μL of 50 × ROX reference dye II, 2 μL of cDNA, and up to 20 μL of H_2_O. Standard curves with threefold dilutions (from a pool consisting of 16 cDNA samples) were generated for each assay, and the amplification efficiency was calculated based on the slopes of the standard curves. The reaction mixtures were incubated in a 96-well plate at 95 °C for 2 min, followed by 45 cycles of 95 °C for 20 s, 60 °C for 35 s and 72 °C for 15 s. The Mx3000/Mx Pro software (Stratagene) was used to construct the melting curve. All reactions were performed in triplicate.

**Table 1:** **Primers used for the qRT-PCR validation of selected miRNA and their target mRNA**

miRNA/mRNA	Primers
ssc-mir-122	GTGGAGTGTGACAATGGTGTTTGA
ssc-mir-451	CGAGGAAACCGTTACCATTACTGAGTT
ssc-mir-504	GGAGACCCTGGTCTGCACTCTATCT
ssc-mir-486	CCTGTACTGAGCTGCCCCGA
ssc-mir-192	CCTGACCTATGAATGACAGCCAAA
5SRNA	GGTTAGTACTTGGATGGGAGACTGCCT
GAPDH-F	TCTTCTGGGTGGCAGTGAT
GAPDH-R	GTTTGTGATGGGCGTGAA
NFAT5-F	AGGGTAGTCGTGGCTCAGTA
NFAT5-R	CAGGGAGTTGTATTTCGCC
NPEPPS-F	AGATGGTGTGTGTGTCCGT
NPEPPS-R	CGTGATGAAGAACAGGAGTT

For the mRNA, the qRT-PCR was performed using the TaKaRa PrimeScript™ RT Reagent Kit and gDNA Eraser (TaKaRa), which comprised a genomic DNA elimination reaction, reverse-transcription reaction and RT-PCR. The PCR primers targeted the exon/exon junctions using DNAMAN 6.2 to avoid possible amplification of any residual genomic DNA, and the specificity was determined using BLASTN. The *GAPDH* gene was used as the internal control, and all primer sequences are shown in Table 1. A melting curve confirmed the specificity of each product, and PCR analyses were performed in triplicate in 20 μL amplification reactions containing 10 μL of 2 × SYBR^®^ Premix Ex Taq™ II, 0.4 μL of ROX II, 2 μL of RT reaction solution (cDNA solution), 6 μL of H_2_O and 0.8 μL (10 mM) of each primer using the following conditions according to the manufacturer’s instructions: 1 cycle of 95 °C for 3 min, 40 cycles of 95 °C for 20 s, 58–61 °C for 20 s and then 72 °C for 15 s. A melting curve analysis (60–95 °C) was performed to assess the amplification specificity.

The relative expression levels of each mRNA and miRNA were calculated using the 2^−ΔΔCT^ method according to the standard curve [22]. Each sample was replicated three times. The levels are expressed as the 2^−ΔΔCT^ means ± standard errors (SEs).

### miRNA target prediction and bioinformatic analysis

The bioinformatic prediction of the miRNA targets was performed using TargetScan Release 6.2 [23]. Due to no database for porcine miRNA in the TargetScan Release 6.2, so the prediction was done using human orthologs of porcine miRNA, assuming that humans and pigs have conserved miRNA-targeting sites at the 3′ UTRs of orthologous mRNA. A functional clustering analysis of the identified miRNA was performed using the Kyoto Encyclopedia of Genes and Genomes (KEGG) pathways database and DIANA-miRPath [24]. Four of the validated up-regulated miRNA (i.e., *ssc*-*miR*-*122*, *ssc*-*miR*-*192*, *ssc*-*miR*-*451* and *ssc*-*miR*-*486*) were selected as the input miRNA for the target gene prediction.

### Cell culture and transfection

PK15 cells were maintained in DMEM (Gibco, USA) supplemented with 10% FBS (Gibco) in a humidified atmosphere of 5% CO2 at 37 °C. The PK15 cells were transfected with the *ssc*-*miR*-*122* mimic and a negative control mimic using lipofectamine 2000 (Invitrogen) according to the manufacturer’s protocol. Transfection complexes were added to the medium at a final oligonucleotide concentration of 30 nM. Similarly, the PK15 cells were transfected with the *ssc*-*miR*-*122* inhibitor or inhibitor negative control at a final oligonucleotide concentration of 100 nM to knockdown *ssc*-*miR*-*122*. The culture medium was replaced 6 h post-transfection with the regular culture medium for 24 h. The miRNA transfection efficiency was verified by qRT-PCR.

### Luciferase reporter assay

To evaluate the interaction between miR-122 and the target genes *nuclear factor of activated T*-*cells 5* (*NFAT5*) and *aminopeptidase puromycin sensitive* (*NPEPPS*), the 3′-untranslated region (3′UTR) sequence of *NFAT5* and *NPEPPS* was constructed into the pGL3-promoter vector (Promega, WI, USA) using the *Xba*I restriction sites. The recombinant plasmids were named NFAT5 3′UTR WT and NPEPPS 3′UTR WT (Figure 5D). The *NFAT5* and *NPEPPS* 3′UTR sequence complementary to the *ssc*-*miR*-*122* seed sequence (GUGA) was then mutated to TGAG and TGAC, and the mutated plasmids were named NFAT5 3′UTR MT and NPEPPS 3′UTR MT (Figure 5D). All above plasmids were confirmed by sequencing. The cells were co-transfected with the reporter construct, an internal control vector (pGL4.74), and a synthetic *ssc*-*miR*-*122* or negative control mimic. Forty-eight hours after the transfection, the luciferase activity was determined using the Dual-Luciferase Reporter Assay System (Promega) according to the manufacturer’s protocol. The relative luciferase expression values were analyzed using a Modulus single-tube multimode reader (Turner BioSystems, CA, USA).

### Western blotting

The total protein was extracted from the PK15 cells using cell lysis buffer for Western blotting (Beyotime, China) with PMSF (Beyotime). The protein concentration of the cell lysate was quantified using a BCA kit (Beyotime), and 40 µg of each protein sample were separated on SDS-PAGE 8% gels and blotted onto a polyvinylidene fluoride (PVFD) membrane (Millipore, USA).The membranes were blocked for 2 h at room temperature in Western Blocking Buffer (Beyotime) and then incubated overnight at 4 °C with rabbit polyclonal antibodies against NFAT5 and NPEPPS (Abcam, USA) at a dilution of 1:1000. To normalize the protein loading, the PVDF membranes were simultaneously incubated with the mouse anti-GAPDH (Beyotime) monoclonal antibody at a dilution of 1:1000. The membranes were washed three times with TBST buffer and then incubated with HRP-conjugated secondary antibodies that were diluted 1000 times at room temperature for 1 h. Finally, the immunoreactive bands were visualized using a DAB Horseradish Peroxidase Color Development Kit (Beyotime).

### Quantification of viral DNA

To determine the effect of *ssc*-*miR*-*122 *on viral protein expression and viral replication, PK15 cells were pretransfected with either *ssc*-*miR*-*122* mimic (30 nM) or mimic control and then infected with PCV2-SD at a multiplicity of infection (MOI) of 0.1 TCID_50_. At 36 h post-infection, the expression levels of the PCV2 Cap protein were analyzed by Western blotting using mouse anti-Cap monoclonal IgG, and the numbers of PCV2 DNA copies were quantified by absolute qRT-PCR, as described below. The viral DNA was extracted from the PK15 cells using a Viral DNA Kit (OMEGA, China) and used for quantifying the copy number of PCV2 genomic DNA by qRT-PCR. The primers (PCV2-RT-F: 5′-CCAGGAGGGCGTTCTGACT-3′ and PCV2-RT-R: 5′-CGTTACCGCTGGAGAAGGAA-3′) were designed according to PCV2-SD sequence. A 99 bp conserved region of the ORF2 gene of PCV2 was amplified by PCR and cloned into a pMD-18T vector (TaKaRa). The resultant plasmid was used as a standard DNA template to optimize the assay conditions. The PCV2 genomic DNA copy number was analyzed by qRT-PCR using the following conditions: 95 °C for 10 s, 95 °C for 5 s, 55 °C for 30 s, 95 °C for 15 s, 60 °C for 30 s and 95 °C for 15 s for 40 cycles. The baseline adjustment method in the MX3000p software (Stratagene) was used to determine the Ct value of each reaction. The copy number in the sample was measured using a linear formula that was established according to the standard curve using the tenfold serial dilution of the standard plasmid. All samples were amplified in triplicate.

### Statistical analysis

The results shown in the figures represent at least three independent trials and are presented as the averages ± SE as indicated. One-way ANOVA and Duncan’s Multiple Range test (SAS version 8.02, 2001) were performed to examine the significance of the differential expression levels among the groups, and the differences among the groups were considered statistically significant at *P* value < 0.05.

## Results

### Sequence analysis of sRNA in porcine lung tissues

The four sRNA libraries of LW-u, LW-i, YL-u and YL-i (each from the pooled lung tissues of four individuals) were constructed and sequenced using an Illumina/Solexa 1G high-throughput sequencer. In total, 9 573 498 (LW-u), 9 570 066 (LW-i), 9 571 684 (YL-u), and 9 573 660 (YL-i) high-quality reads were obtained. After removing adaptors and insufficient tags, 9 440 912 (LW-u), 9 455 761 (LW-i), 9 511 998 (YL-u), and 9 533 777 (YL-i) clean reads of 18-30 nt were ultimately retained (Table 2). Amongst these reads, 61.04% (LW-u), 65.22% (LW-i), 64.41% (YL-u), and 63.39% (YL-i) of the total reads were perfectly mapped to the *Sus scrofa* genome (Additional file 1). Most sRNA were 19–24 nt in length. The 22-nt long sRNA sequences were the most abundant and accounted for more than 50% of the sRNA, followed by the 23-nt and 21-nt long sRNA, which is typical of sRNA Dicer-processed products and is consistent with the known 18–25 nt range of miRNA (Additional file 2). To assess the sRNA detection efficiency of the high-throughput sequencing, all sequence reads were annotated and classified by aligning the sequence reads to sequences in the GenBank and Rfam databases. Consequently, 35 332 (LW-u), 45 265 (LW-i), 48 905 (YL-u), and 53 405 (YL-i) (15.72, 15.95, 16.52, and 16.84%, respectively) unique sRNA were annotated in the four libraries.

**Table 2:** **Summary of clean reads produced by the sRNA sequencing after filtering the contaminated reads**

Type	LW-u		LW-i		YL-u		YL-i	
	Amount	Percent (%)	Amount	Percent (%)	Amount	Percent (%)	Amount	Percent (%)
Total_reads^a^	9 600 000		9 597 432		9 600 000		9 600 000	
High_quality^b^	9 573 498	100	9 570 066	100	9 571 684	100	9 573 660	100
3′adapter_null^c^	1308	0.01	1405	0.01	1626	0.02	1307	0.01
Insert_null^d^	2740	0.03	2833	0.03	1254	0.01	1075	0.01
5′adapter_contaminants^e^	108 280	1.13	71 737	0.75	35 436	0.37	26 874	0.28
Smaller_than_18nt^f^	20 206	0.21	38 263	0.40	21 318	0.22	10 545	0.11
PolyA^g^	52	0.00	67	0.00	52	0.00	82	0.00
Clean_reads^h^	9 440 912	98.62	9 455 761	98.81	9 511 998	99.38	9 533 777	99.58

^a^Total reads: total sequenced reads, which are generally required to be > 5 million.

^b^High_quality: number of high-quality reads.

^c^3′adapter_null: number of reads with no 3′ adaptor.

^d^Insert_null: number of reads with no insertion.

^e^5′adapter_contaminants: number of 5′ contaminants.

^f^Smaller_than_18 nt: number of reads less than 18 nt.

^g^PolyA: number of reads with polyA.

^h^Clean_reads: number of clean reads after removing the adaptors and contaminants. Clean reads were used in all analyses in this study.

### Known conserved and differentially expressed miRNA in porcine lung tissues

**Figure 1:**
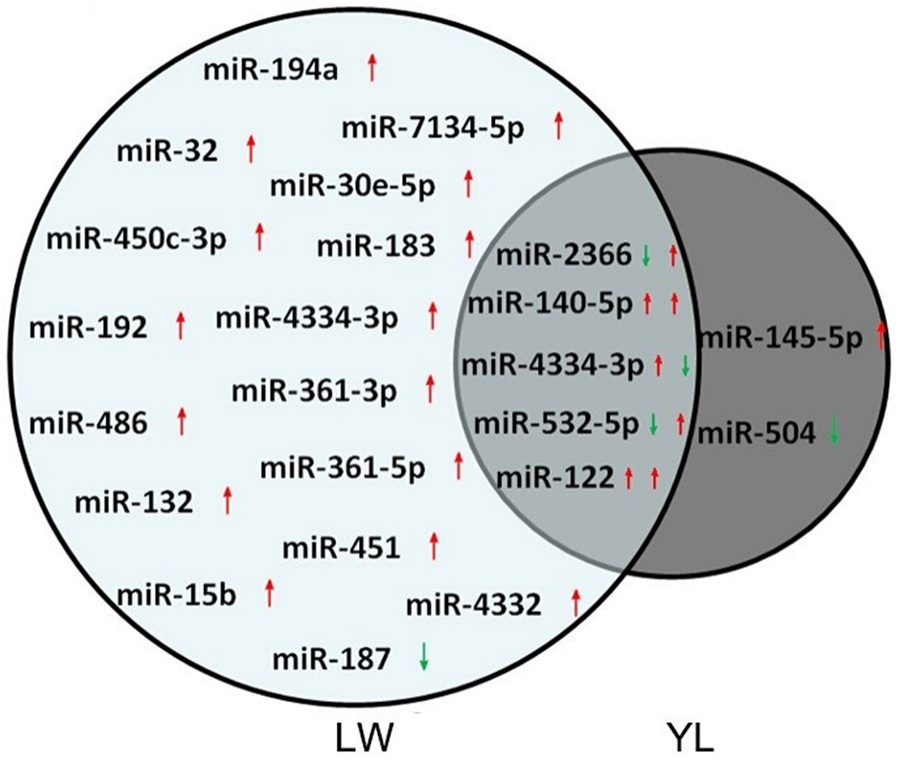
**Venn diagram showing significantly differentially expressed known miRNA in the LW-i vs.** **LW-u** **and/or YL-i** **vs.** **YL-u pigs.** Eighteen miRNA were significantly up-regulated, and three miRNA were significantly down-regulated in the LW-i vs. LW-u pigs, while five miRNA were significantly up-regulated and two miRNA were significantly down-regulated in the YL-i vs. YL-u pigs. Both *ssc*-*miR*-*122* and *ssc*-*miR*-*140*-*5p* were up-regulated in both breeds, and the expression of *ssc*-*miR*-*532*-*5p*, *ssc*-*miR*-*2366* and *ssc*-*miR*-*4334*-*3p* changed in the opposite direction in different breeds.*↑* up-regulated miRNA, *↓* down-regulated miRNA.

The BLASTN searches (number of mismatches ≤ 3) and further sequence analyses showed that 5 404 421 (LW-u), 5 484 886 (LW-i), 5 358 244 (YL-u), and 5 279 602 (YL-i) sRNA sequences perfectly matched known pig, human and mouse miRNAome sequences, corresponding to 295 unique known miRNA across the four libraries. Among the conserved miRNA, *ssc*-*let*-*7a*, *ssc*-*let*-*7f*, *ssc*-*let*-*7c*, *ssc*-*let*-*7e*, *ssc*-*miR*-*103*, *ssc*-*miR*-*140*-*3p*, *ssc*-*miR*-*199a*-*3p* and *ssc*-*miR*-*199b*-*3p* had more than 100 000 reads. The 10 most abundantly co-expressed miRNA in the LW and YL groups are listed in Table 3. In LW pigs, 18 miRNA were significantly up-regulated (*P* < 0.05; fold-change of < −1 or > 1) after PCV2 infection, and three miRNA were significantly down-regulated. In YL pigs, five miRNA were significantly up-regulated, and two miRNA were significantly down-regulated (Figure 1 and Table 4). The relative abundances of the miRNA in the uninfected and infected pairs showed that the majority of the differentially expressed miRNA had fold changes ranging from −2 to 5. Of the up-regulated miRNA, *ssc*-*miR*-*122* had the highest fold change (4.95), while of the down-regulated miRNA, *ssc*-*miR*-*532*-*5p* had the highest fold-change (−2.2) (Table 4). Hierarchical clustering analysis based on the relative expression frequencies of the miRNA in the LW and YL groups suggests that the miRNA expression levels were different between LW-u and LW-i and between YL-u and YL-i (Additional file 3).

**Table 3:** **Ten most abundantly co-expressed miRNA in porcine lung tissues**

Mir-name^a^	LW-i-std^b^	LW-u-std	Fold-change^c^	*P* value^d^	Sig-label^e^	YL-i-std	YL-u-std	Fold-change	*P* value	Sig-label
ssc-let-7a	200 116.9	223 606.8	−0.160121	0	#	190 431.98	205 049.8	−0.106698	0	#
ssc-let-7f	153 283.9	147 584.8	0.05466	7.91E−224	#	154 279.99	162 951.5	−0.078891	0	#
ssc-let-7c	23 084.45	26 634.82	−0.206393	0	#	25 229.77	25 631	−0.022763	4.02E−08	#
ssc-miR-140-3p	19 191.47	14 749.21	0.37983	0	#	27 233.803	13 713.84	0.98977	0	#
ssc-miR-103	18 098.7	15 669.88	0.20789	0	#	13 130.368	13 746.64	−0.066172	4.09E−31	#
ssc-let-7e	13 082.61	16 618.84	−0.34517	0	#	12 454.455	13 656.54	−0.13293	1.15E−116	#
ssc-miR-199a-3p	11 877.63	10 953.92	0.1168	8.59E−79	#	10 987.566	12 309.09	−0.163852	2.36E−157	#
ssc-miR-199b-3p	11 877.52	10 953.92	0.11679	8.94E−79	#	10 987.461	12 308.88	−0.163841	2.48E−157	#
ssc-let-7g	10 709.87	9836.656	0.1227	2.92E−78	#	9316.1399	10 257.47	−0.13887	8.59E−96	#
ssc-miR-107	10 397.37	8750.532	0.24878	2.47E−293	#	7124.6684	7155.805	−0.006291	0.421371	#

^a^miR-name: miRNA name.

^b^std: normalized expression level of miRNA in each sample.

^c^Fold-change = log_2_ (treatment/control).

^d^*P* value: the significance of the differential miRNA expression between the samples; low *P* values indicating higher significance of miRNA expression.

^e^Sig-labe

#: no significant difference.

**Table 4:** **Significantly differentially expressed miRNA in the lung tissues from LW and YL pigs**

MiR-name	LW-i-std	LW-u-std	Fold-change	*P* value	Sig-label^b^	YL-i-std	YL-u-std	Fold-change	*P* value	Sig-label
ssc-miR-122	9434.249	304.42	4.95377399	0	**	1236.551	553.72	1.15908854	0	**
ssc-miR-192	911.5078	91.728	3.31281462	0	**	144.7485	105.97	0.44987344	3.72E−14	#
ssc-miR-450c-3p	1.3748	0.2118	2.69844726	0.0042214	**	–^a^	–	–	–	
ssc-miR-194a	7.0856	1.8007	1.97633218	2.27E−08	**	1.5734	1.2616	0.31863092	0.575742	#
ssc-miR-486	92.7477	27.434	1.75735703	6.45E−79	**	39.4387	20.816	0.92193299	8.90E−14	#
ssc-miR-183	3.1727	0.9533	1.73470891	0.0006918	**	1.4685	1.577	−0.10283939	0.850583	#
ssc-miR-32	4.1245	1.2711	1.6981417	0.0001305	**	6.1885	5.2565	0.23548725	0.397596	#
ssc-miR-144	37.649	11.651	1.69210823	2.07E−31	**	25.3834	13.667	0.89319929	5.85E−09	#
ssc-miR-4334-3p	1.9036	0.7415	1.36021176	0.0292532	*	1.6782	4.4155	−1.39566215	0.000566	**
ssc-miR-132	50.3397	20.125	1.3226935	2.76E−29	**	36.5018	24.39	0.58166602	1.58E−06	#
ssc-miR-30e-3p	39.5526	15.994	1.3062237	6.21E−23	**	13.1113	19.134	−0.54530515	0.001051	#
ssc-miR-451	1706.261	697.18	1.29123891	0	**	960.7944	483.39	0.99104161	0	#
ssc-miR-4332	3.0669	1.2711	1.27070359	0.0080352	**	3.776	4.2052	−0.15531565	0.641569	#
ssc-miR-140-5p	3.4899	1.4829	1.23476439	0.0056075	**	5.6641	1.9975	1.50365123	3.52E−05	**
ssc-miR-15b	171.1126	74.463	1.20034841	2.99E−82	**	173.4884	109.76	0.66053808	8.63E−32	#
ssc-miR-361-5p	16.8151	7.4145	1.18133605	2.99E−09	**	24.7541	17.347	0.5130229	0.000421	#
ssc-miR-7134-5p	22.8432	10.486	1.12325904	3.03E−11	**	23.4954	22.498	0.06258799	0.650382	#
ssc-miR-361-3p	4.1245	2.0125	1.03523045	0.0087951	**	2.4125	2.6283	−0.12360102	0.769294	#
ssc-miR-532-5p	15.8633	72.768	−2.1976	3.31E−83	**	50.4522	13.877	1.86220068	1.46E−47	**
ssc-miR-2366	0.3173	1.377	−2.1176	0.0126121	*	1.4685	0.5257	1.48203163	0.041899	*
ssc-miR-187	0.9518	2.754	−1.5328	0.0038754	**	1.2587	1.3667	−0.11876213	0.840414	#
ssc-miR-145-5p	743.9909	501.12	0.57013807	5.80E−100	#	3461.482	1102.6	1.65047065	0	**
ssc-miR-504	5.7108	6.0376	−0.08028232	0.7705841	#	3.3565	7.0437	−1.06937582	0.000392	**

^a^Normalized expression was less than 1 in both samples and these reads were removed from further analysis.^b^Sig-label: **: fold-change > 1 or < −1, and *P* value < 0.01; *: fold-change > 1 or < −1, and *P* value < 0.05; #: no significant difference.

### Identification of novel miRNA candidates

In this study, 3 563 675 (LW-u), 3 130 638 (LW-i), 3 298 682 (YL-u), and 3 261 707 (YL-i) unannotated sRNA, representing 137 325 (LW-u), 168 605 (LW-i), 169 308 (YL-u), and 179 586 (YL-i) unique sRNA, were mapped to the *Sus scrofa* reference genome. To determine whether these sRNA sequences were genuine porcine miRNA, we investigated their hairpin structures, Dicer cleavage sites, and minimal free energies using MIREAP v0.2 [25]. Mfold [26] and MiPred [27] to predict the typical secondary structures of the miRNA precursors and identify the pseudo-pre-miRNA. In total, 95 potential novel miRNA candidates with lengths ranging from 20 to 24 nt and numbers of reads ranging from 5 to 3968 were obtained from the four libraries (Additional files 4 and 5). In LW pigs, seven novel miRNA were significantly up-regulated (*P* < 0.01; fold-change of < −1 or > 1) and five novel miRNA were significantly down-regulated after PCV2 infection. In YL pigs, seven novel miRNA were significantly up-regulated, and nine novel miRNA were significantly down-regulated. Of these differentially expressed novel miRNA, novel-mir-25 was up-regulated and novel-mir-60 was down-regulated in both breeds, while the expression of novel-mir-63 changed in the opposite direction in different breeds (Additional file 4).

### Validation of differentially expressed miRNA and affected pathways

**Figure 2:**
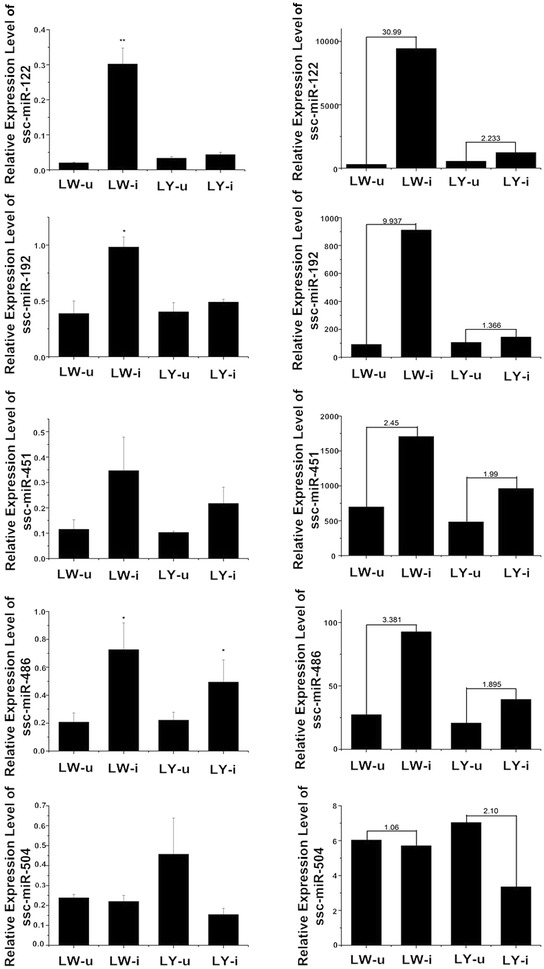
**Five differentially expressed miRNA in porcine lung tissues were validated by qRT-PCR.** The order of miRNA shown from top to bottom is *ssc*-*miR*-*122*, *ssc*-*miR*-*192*, *ssc*-*miR*-*451*, *ssc*-*miR*-*486* and *ssc*-*miR*-*504*. The Solexa sequencing results are shown on the right, and the qRT-PCR results are shown on the left. * and ** indicate significance at the *P* value threshold levels of 0.05 and 0.01, respectively. The expression levels of three of the five miRNA (except for *ssc*-*miR*-*451* and *ssc*-*miR*-*504*) were consistent with the sequencing results with *P* < 0.05.

The qRT-PCR validation of the differentially expressed miRNA indicated that five miRNA (i.e., *ssc*-*miR*-*122*, *ssc*-*miR*-*192*, *ssc*-*miR*-*451,ssc*-*miR*-*486* and *ssc*-*miR*-*504*) had differential expression patterns that were similar to the sRNA sequencing data (Figure 2). Of these miRNA, *ssc*-*miR*-*122* and *ssc*-*miR*-*192* had significantly higher expression levels in the PCV2-infected LW pigs than those in the uninfected LW pigs, while the expression of *ssc*-*miR*-*486* in both breeds of PCV2-infected pigs was significantly higher than that in the uninfected pigs. The TargetScan analyses using these miRNA predicted significantly conserved genes in several KEGG pathways, including the insulin signaling pathway [nine predicted target genes, −In(*P* value) = 7.75], the mTOR signaling pathway [four genes, −In(*P* value) = 4.75], non-small cell lung cancer (NSCLC) [four genes, −In(*P* value) = 3.71], and leukocyte transendothelial migration [six genes, −In(*P* value) = 3.61] (Additional file 6).

### Expression profile of *miR*-*122* in different tissues

**Figure 3:**
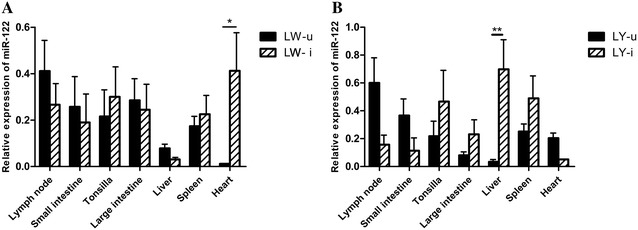
**Changes in the expression of** ***miR*-*122*** **in the different tissues from LW (A) and YL (B) pigs after PCV2 infection.** The *y*-axis indicates the fold changes in normalized expression. SEs from the mean are labeled on the bar using vertical lines. * and ** indicate significance at the *P* value threshold levels of 0.05 and 0.01, respectively.

*MiR*-*122* is a broad-range miRNA that can express in many tissues; therefore, we assessed the expression profile of *miR*-*122* in different tissues, including the lymph node, small intestine, tonsil, large intestine, liver, spleen and heart, in the LW and YL pigs after the PCV2 infection. A comparison of the PCV2-infected pigs and PCV2-uninfected pigs indicates that the expression of *miR*-*122* was significantly higher in the hearts of the infected LW pigs (*P* < 0.05) and the livers of the infected YL pigs (*P* < 0.01) (Figure 3). Meanwhile, in both pig breeds, the expression of *miR*-*122* was higher in the tonsil and spleen and lower in the lymph node and small intestine of the infected pigs than that in the uninfected pigs, but this pattern was not statistically significant (*P* > 0.05) (Figure 3).

### *MiR*-*122* can repress the protein expression and viral DNA replication of PCV2

**Figure 4:**
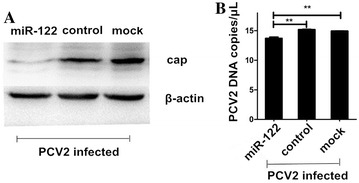
**The expression of Cap protein (A) and viral DNA copies (B) in PCV2-infected PK15 cells transfected with** ***ssc*-*miR*-*122***. PK15 cells were pretransfected with *ssc*-*miR*-*122* mimic and mimic control and then infected with PCV2 at an MOI of 0.1. Both the total protein and viral DNA were harvested from the PK15 cells 36 h post-infection to analyze the expression of Cap protein and viral DNA copies. In panel B, SE from the mean are labeled on the bar using vertical lines. ** Indicates significance at the *P* value threshold level of 0.01.

As mentioned in materials and methods, PK15 cells were pretransfected with either *ssc*-*miR*-*122* mimic or mimic control and then infected with PCV2. At 36 h post-infection, the expression levels of the PCV2 Cap protein and the copy numbers of PCV2 DNA were quantified. As shown in Figure 4, the Cap protein expression and viral DNA copies were significantly reduced when the cells were transfected with *ssc*-*miR*-*122* compared to those in the mimic-control-treated infected cells and untreated infected cells (*P* < 0.01). Thus, *ssc*-*miR*-*122* could decrease PCV2 viral DNA replication and protein synthesis in PK15 cells.

### Nuclear factor of activated T-cells 5 (NFAT5) and aminopeptidase puromycin sensitive (NPEPPS) were directly targeted by miR-122

**Figure 5:**
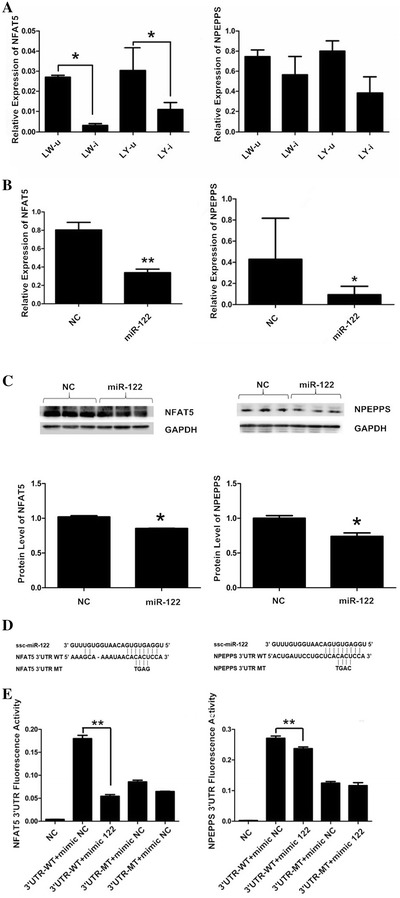
**Validation of** ***NFAT5*** **and** ***NPEPPS*** **as direct targets of** ***ssc*-*miR*-*122***. **A** mRNA expression of *NFAT5* and *NPEPPS* in lung tissues from uninfected or infected LW and YL pigs. **B** mRNA expression of *NFAT5* and *NPEPPS* in PK15 cells transfected with the *ssc*-*miR*-*122* mimic. **C** Protein expression of NFAT5 and NPEPPS in PK15 cells transfected with *ssc*-*miR*-*122* mimic. **D** Diagram of the predicted *miR*-*122* targeting site within the 3′ UTR of *NFAT5* and *NPEPPS* (3′ UTR WT). The mutated 3′ UTR (3′ UTR MT) contains a mutated sequence which is not complementary to the *ssc*-*miR*-*122* seed sequence (GUGA).**E** Relative fluorescence activity of the luciferase constructs harboring the wildtype or mutant 3′ UTR of either *NFAT5* or *NPEPPS* genes in PK15 cells with or without the *ssc*-*miR*-*122* mimic. SE from the mean are labeled on the bar using vertical lines. *, ** and *** indicate significance at the *P* value threshold levels of 0.05, 0.01 and 0.001, respectively.

Since the expression of *miR*-*122* was greatly increased in the LW pigs but remained much lower in the YL pigs after the PCV2 infection, the target genes of *miR*-*122* in PCV2 infection were further analyzed. According to the bioinformatic predictions, *miR*-*122* had a high complementarity and binding specificity with putative binding sites within the 3′ UTR of the porcine *NFAT5* and *NPEPPS* genes. To confirm the relationship between *miR*-*122* and the *NFAT5* and *NPEPPS* genes, we first measured the expression levels of *NFAT5* and *NPEPPS* in the lung tissue. *NFAT5* was significantly down-regulated in the PCV2-infected pigs compared with that in the uninfected pigs (*P* < 0.05), which was the opposite of the expression levels of *miR*-*122* (Figure 5A). The changes in the expression of *NPEPPS* were similar to those observed in *NFAT5* (Figure 5A). The transfection of *ssc*-*miR*-*122* into the PK15 cells markedly decreased the mRNA (*P* < 0.01) and protein levels of *NFAT5* (*P* < 0.05), and mRNA and protein levels of *NPEPPS* (*P* < 0.05) (Figures 5B and C). Next, we verified that *NFAT5* and *NPEPPS* are the direct targets of *ssc*-*miR*-*122* using the vector pGL3-promoter linked to wild or mutant 3′ UTRs of porcine *NFAT5* and *NPEPPS* genes, respectively (Figure 5D). The co-transfection of the luciferase constructs with the *ssc*-*miR*-*122* mimic into the PK15 cells showed that the *ssc*-*miR*-*122* mimic significantly decreased the luciferase activity from the luciferase constructs harboring the wildtype 3′ UTR of both *NFAT5* and *NPEPPS* but not of the mutant 3′ UTR of the two genes (*P* < 0.001) (Figure 5E). Altogether, these results indicate that both the *NFAT5* and *NPEPPS* genes are regulated by *ssc*-*miR*-*122* through binding to their 3′ UTR.

## Discussion

The susceptibility to PCV2 infection differs among pig breeds. According to our previous study, PCV2-infected YL pigs show serious lesions in lung tissues, while infected LW pigs exhibit no such clinical characteristics. Using RNA-seq, the differentially expressed genes between infected and uninfected LW and YL pigs, including *SERPINA1*, were identified in the lung tissues [13]. The PCV2 DNA does not express miRNA in subclinically infected tonsils and mediastinal lymph nodes; however, after the PCV2 infection, eight differentially expressed miRNA encoded by the porcine genome were detected in mediastinal lymph nodes [12]. Furthermore, an in vitro PCV2 infection study using the PK15 cells also detected tens of miRNA induced by the expression of PCV2-encoded ORF1, ORF2 and ORF3 proteins [11]. Due to the involvement of miRNA in PCV2 infection, we compared the miRNA expression profiles in the lung tissues of infected and uninfected LW and YL pigs. In the present study, 295 known porcine miRNA were detected in the porcine lung tissues, and more differentially expressed miRNA were detected in the LW pigs than in the YL pigs after the PCV2 infection. Of these miRNA, *miR*-*132*, *miR*-*187* and *miR*-*451* were also detected in the PK15 cells expressing the PCV2-encoded ORF proteins [11]. Moreover, 95 novel miRNA candidates were identified for the first time.

In this study, the expression patterns of five differentially expressed miRNA (i.e., *ssc*-*miR*-*122*, *ssc*-*miR*-*451*, *ssc*-*miR*-*486*, *ssc*-*miR*-*504* and *ssc*-*miR*-*192*) were validated using sRNA sequencing data. Of these miRNA, *ssc*-*miR*-*122*, *ssc*-*miR*-*451*, *ssc*-*miR*-*486* and *ssc*-*miR*-*192* were up-regulated after the PCV2 infection, while the expression level of *ssc*-*miR*-*504* was down-regulated. By analyzing sRNA libraries constructed from mediastinal lymph nodes of PCV2-infected and uninfected pigs, Núñez-Hernández et al. detected five up-regulated and three down-regulated miRNA in PCV2-infected pigs [12]. However, none of the eight differentially expressed miRNA were found in our study investigating PCV-infected lung tissues. This difference is likely due to the tissue-specific nature of miRNA expression. However, the mTOR and leukocyte transendothelial migration signaling pathways associated with the four up-regulated miRNA in our study were also reported by Núñez-Hernández et al. [12], although the miRNA affected by PCV2 were different.

Of the four up-regulated miRNA, *miR*-*122* was further analyzed because its expression is greatly stimulated in LW pigs after PCV2 infection, while it is stable in YL pigs. Porcine *miR*-*122* was first identified to be specifically expressed in the liver tissue [28, 29]. Subsequently, *miR*-*122* was also found to be expressed in the porcine backfat [30] and *longissimus dorsi* muscle tissues. In intestinal samples collected from pigs during hypothermic circulatory arrest, the expression of *miR*-*122* was significantly increased [31]. In an ischemic porcine cardiogenic shock model, therapeutic hypothermia significantly reduced the levels of *miR*-*122* [32], and plasma *miR*-*122* was associated with acute coronary syndrome [33]. In this study, *miR*-*122* was expressed in the porcine lung tissue. These data collectively indicate that *miR*-*122* is also expressed in tissues other than the liver.

Regarding the relationship between *miR*-*122* and viral infection, most studies have focused on human hepatitis C virus (HCV). In humans, *miR*-*122* is reported to enhance the accumulation of HCV RNA by binding to the 5′ UTR of the HCV genome [34]. Moreover, the 3′ region of the HCV genome targeted by *miR*-*122* is involved in regulating different steps of the HCV replication cycle [35]. Our results also indicate that *ssc*-*miR*-*122* could decrease PCV2 viral DNA replication and protein synthesis in PK15 cells. We subsequently analyzed the 1.7 kb genomic sequence of porcine PCV2 virus and did not find binding sites for *ssc*-*miR*-*122*, suggesting that *miR*-*122* cannot directly interfere with the replication of PCV2 virus by targeting genes encoded by the PCV2 virus. We further examined host genes that are likely targets of *miR*-*122* and found that *NFAT5* and *NPEPPS* were negatively regulated by *miR*-*122* through binding to their 3′ UTR regions.

NFAT5 is a member of the NFAT family of transcription factors and regulates gene expression induced by osmotic stress in mammalian cells. NFAT5 was shown to be up-regulated in lung adenocarcinoma cells, and the knockdown of NFAT5 decreased the proliferation and migration of these cells [36]. In this study, after the PCV2 infection, the NFAT5 expression was decreased due to the increased expression of *miR*-*122* in the lung tissue, which is consistent with the lesions observed in the lung tissue after the PCV2 infection [13]. In humans, NFAT5 plays a crucial role in the regulation of HIV-1 replication by directly interacting with a highly conserved long terminal repeat site and the viral promoter [37] and is also modulated by nonstructural 5A for HCV propagation [38]. *NPEPPS* encodes puromycin-sensitive aminopeptidase, which is a zinc metallopeptidase that hydrolyzes amino acids from the N-terminus of its substrate. In the lung cancer cell line PGCL3, *miR*-*614* inhibited lung cancer cell invasion and proliferation by targeting the 3′ UTR of the *NPEPPS* gene [39]. Further investigations are needed to determine whether NFAT5 and NPEPPS play direct roles in the regulation of PCV2 replication.

In conclusion, the miRNA profile in the porcine lung tissue of PCV2-infected and PCV2-uninfected LW and YL pigs was compared. We identified 21 and seven differentially expressed known miRNA in the lung tissues of LW and YL pigs that were related to the PCV2 infection. The expression levels of *ssc*-*miR*-*122*, *ssc*-*miR*-*451*, *ssc*-*miR*-*486*, and *ssc*-*miR*-*192* were elevated in the PCV2-infected pigs compared to those in the uninfected pigs, while that of *ssc*-*miR*-*504* was reduced. In the PK15 cells, *ssc*-*miR*-*122* can repress the protein expression and viral DNA replication of PCV2, and *ssc*-*miR*-*122* may down-regulate the expression of *NFAT5* and *NPEPPS* by binding to their 3′ UTR.

## Additional files

**Additional file 1.** **Percentages of small non-coding RNA mapped to the** ***Sus scrofa*** **reference genome.** ^a^ Number of clean reads.

**Additional file 2.** **Length distribution and abundance of sequences in LW-u (A), LW-i (B), YL-u (C) and YL-i (D) pigs**. The most abundant lengths were 22 nt, followed by 23 nt, 21 nt and 24 nt.

**Additional file 3.** **Hierarchical clustering of miRNA expression.** This analysis clustered miRNA with similar patterns of expression. Each row represents a specific miRNA, and each column represents a pair of samples. That is, the color of each lattice shows the difference in the expression of a given miRNA between a pair of samples. Take LW-u vs. LW-i for example; the red indicates that the expression of a given miRNA in LW-u is higher than that of LW-i; the green indicates that the expression of a given miRNA in LW-u is lower than that of LW-i; and the grey indicates that a given miRNA is not expressed in at least one sample.

**Additional file 4.** **Information of the novel miRNA in porcine lung tissues.** This excel file contains all information of the 95 novel miRNA, including mature miRNA sequence, precursor sequence, precursor location, minimal free energy, normalized expression value, fold-change and *P* value. ^a^ If the normalized expression of a given miRNA was less than 1 in both samples of a sample pair, the normalized expression of both samples were not shown and the miRNA was removed from the differential expression analysis. ^b^ Sig-label: **: fold-change > 1 or < −1, and *P* value < 0.01.

**Additional file 5.** **Secondary structures of the novel miRNA.**

**Additional file 6.** **DIANA-miRPath predicted KEGG pathways of four up-regulated miRNA.** The *y*-axis indicates the confidence level that a differential expressed miRNA is enriched to a certain KEGG pathway. Confidence levels were measured by –In(*P-*value) and correlated positively. Union group indicates the pathway analysis of a combination of all four miRNA.

## Abbreviations

PCV2: porcine circovirus type 2
PCV1: porcine circovirus type 1
PRRSV: porcine reproductive and respiratory syndrome virus
PPV: porcine parvovirus
HCV: hepatitis C virus
miRNA: microRNA
sRNA: small RNA
LW pig: Laiwu pig
YL pig: Yorkshire × Landrace crossbred pig
LW-i: PCV2-infected LW pig
LW-u: PCV2-uninfected LW pig
YL-i: PCV2-infected YL pig
YL-u: PCV2-uninfected YL pig
NFAT5: nuclear factor of activated T-cells 5
NPEPPS: aminopeptidase puromycin sensitive
3′UTR: 3′untranslated region
qRT-PCR: quantitative real-time PCR
RNA-seq: RNA-sequencing
MOI: multiplicity of infection
TCID_50_: 50% tissue culture infective dose
SE: standard error
