# Supplementary material for: Identification and characterization of microRNA in the lung tissue of pigs with different susceptibilities to PCV2 infection
Source: Vet Res. 2018 Feb 15;49:18. doi: 10.1186/s13567-018-0512-3 (PMC5815207; doi:10.1186/s13567-018-0512-3)
Supplement: Supplementary file 1 — Additional file 1. Percentages of small non-coding RNA mapped to the Sus scrofa reference genome. a Number of clean reads. [file 13567_2018_512_MOESM1_ESM.docx]

|  |  | **Unique sRNAs** | **Percent** | **Total sRNAs** | **Percent** |
| --- | --- | --- | --- | --- | --- |
| LW-u | snRNAs | 224855 ^a^ | 100% | 9440912 | 100% |
|  | mapped snRNAs | 111454 | 49.57% | 5763039 | 61.04% |
| LW-i | snRNAs | 283728 | 100% | 9455761 | 100% |
|  | mapped snRNAs | 142691 | 50.29% | 6167293 | 65.22% |
| YL-u | snRNAs | 295897 | 100% | 9511998 | 100% |
|  | mapped snRNAs | 155610 | 52.59% | 6126551 | 64.41% |
| YL-i | snRNAs | 317234 | 100% | 9533777 | 100% |
|  | mapped snRNAs | 168782 | 53.20% | 6043588 | 63.39% |
