# Supplementary figures and images for: Identification and characterization of microRNA in the lung tissue of pigs with different susceptibilities to PCV2 infection
Source: Vet Res. 2018 Feb 15;49:18. doi: 10.1186/s13567-018-0512-3 (PMC5815207; doi:10.1186/s13567-018-0512-3)

**
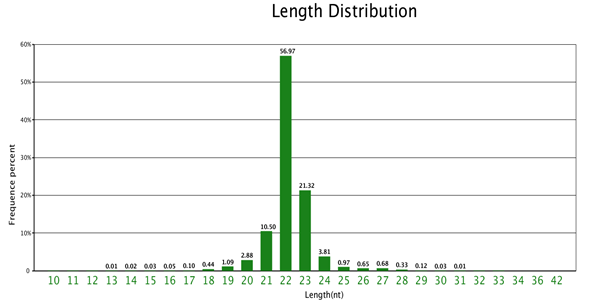
**

**A**


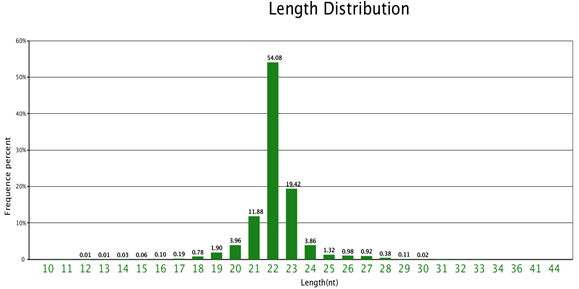


**B**


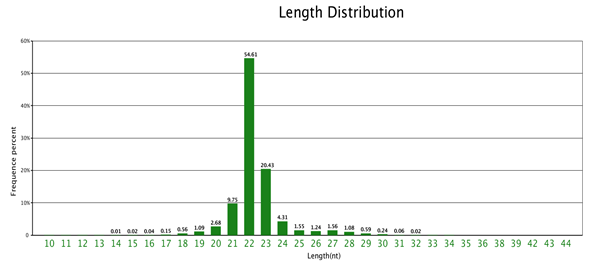


**C**


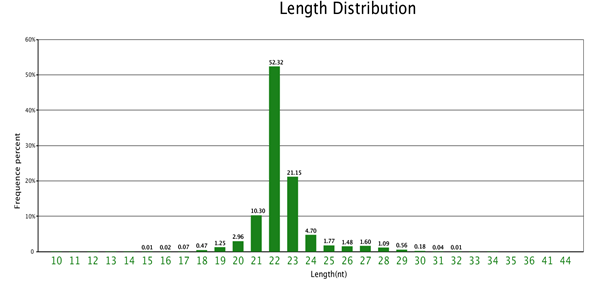


**D**

Supplement: Supplementary file 2 — Additional file 2. Length distribution and abundance of sequences in LW-u (A), LW-i (B), YL-u (C) and YL-i (D) pigs. The most abundant lengths were 22 nt, followed by 23 nt, 21 nt and 24 nt. [file 13567_2018_512_MOESM2_ESM.docx]

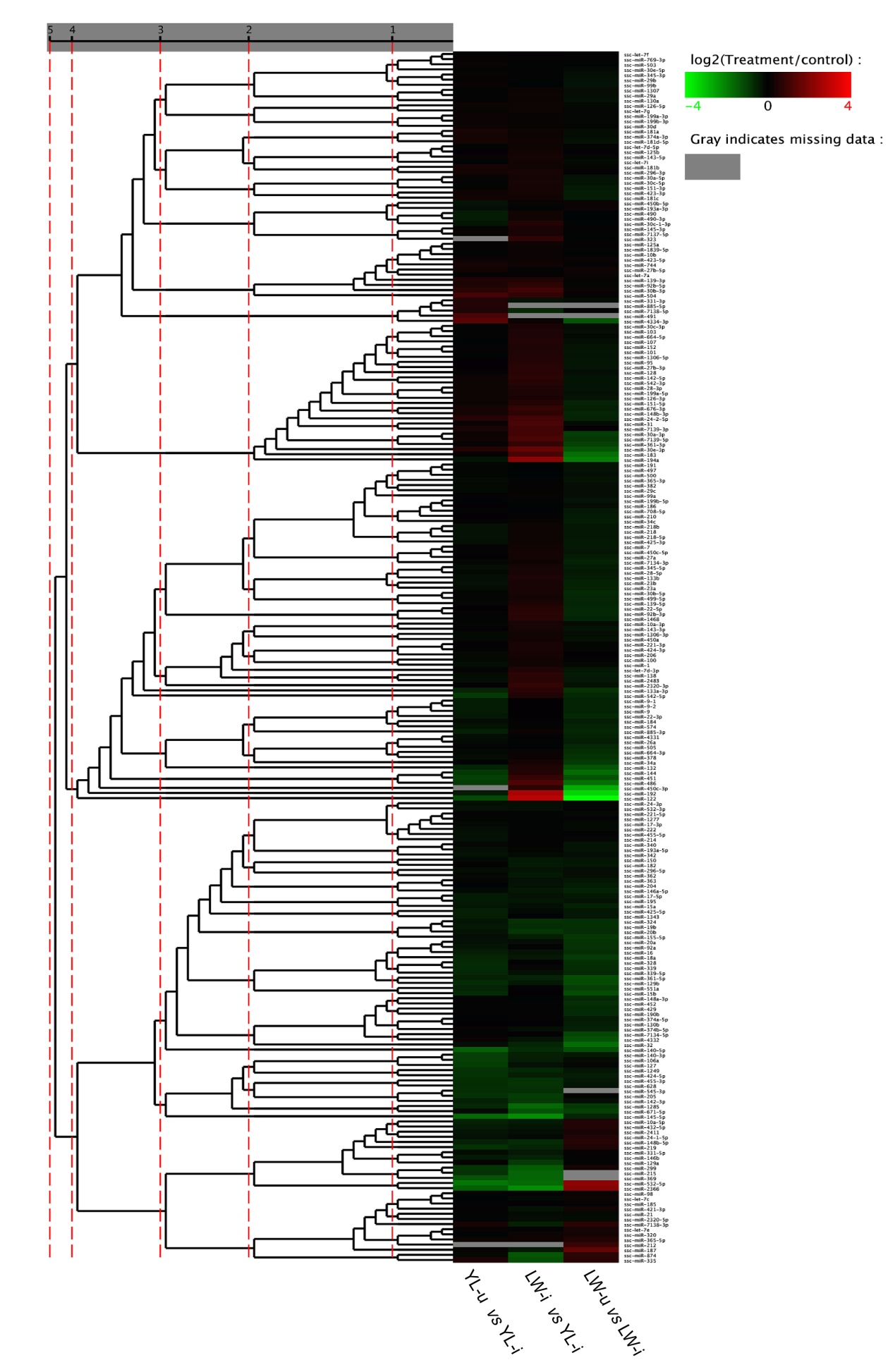

Supplement: Supplementary file 3 — Additional file 3. Hierarchical clustering of miRNA expression. This analysis clustered miRNA with similar patterns of expression. Each row represents a specific miRNA, and each column represents a pair of samples. That is, the color of each lattice shows the difference in the expression of a given miRNA between a pair of samples. Take LW-u vs. LW-i for example; the red indicates that the expression of a given miRNA in LW-u is higher than that of LW-i; the green indicates that the expression of a given miRNA in LW-u is lower than that of LW-i; and the grey indicates that a given miRNA is not expressed in at least one sample. [file 13567_2018_512_MOESM3_ESM.docx]

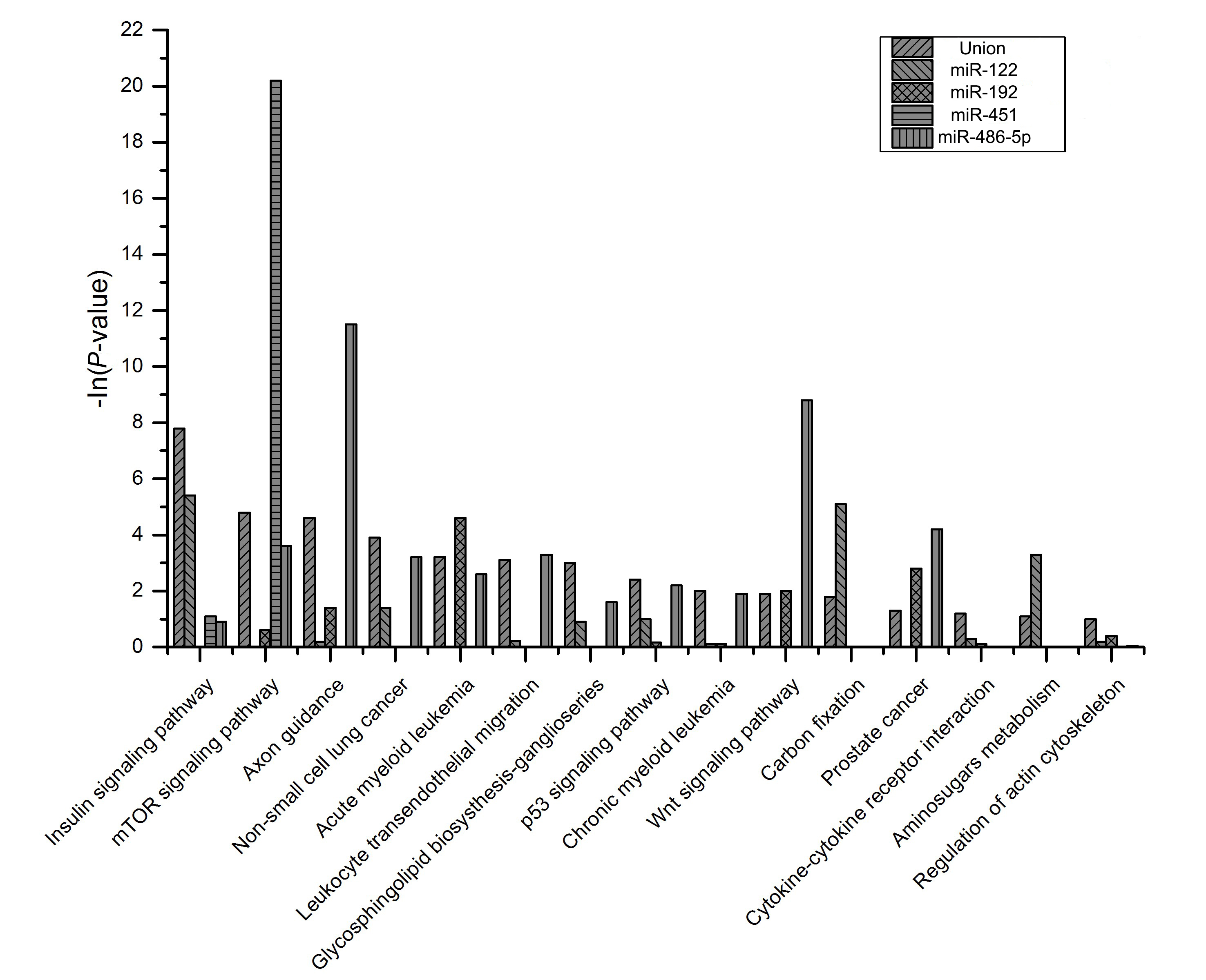

Supplement: Supplementary file 6 — Additional file 6. DIANA-miRPath predicted KEGG pathways of four up-regulated miRNA. The y-axis indicates the confidence level that a differential expressed miRNA is enriched to a certain KEGG pathway. Confidence levels were measured by –In(P-value) and correlated positively. Union group indicates the pathway analysis of a combination of all four miRNA. [file 13567_2018_512_MOESM6_ESM.jpg]
