# Supplementary material for: Identification and characterization of microRNA in the lung tissue of pigs with different susceptibilities to PCV2 infection
Source: Vet Res. 2018 Feb 15;49:18. doi: 10.1186/s13567-018-0512-3 (PMC5815207; doi:10.1186/s13567-018-0512-3)
Supplement: Supplementary file 5 — Additional file 5. Secondary structures of the novel miRNA. [file 13567_2018_512_MOESM5_ESM.doc]

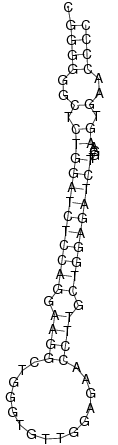

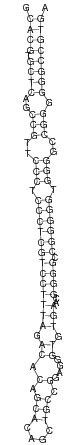

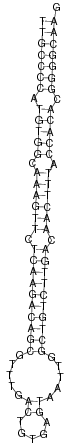

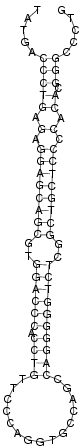

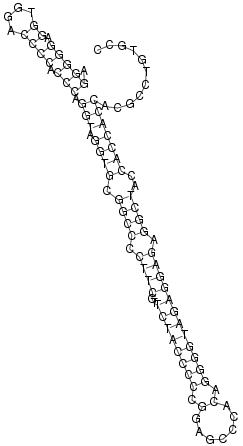

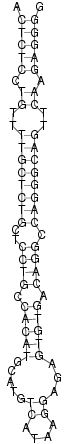

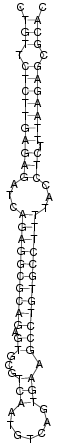

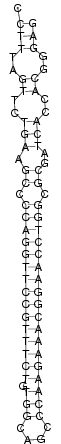


**The secondary structures above are arranged from left to right in the following order: novel_miR_1, novel_miR_2, novel_miR_3, novel_miR_5, novel_miR_6, novel_miR_7, novel_miR_8 and novel_miR_9.**


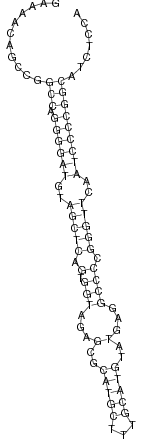

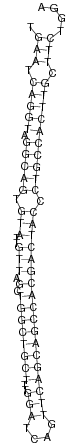

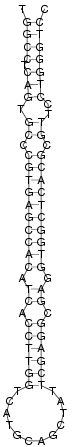

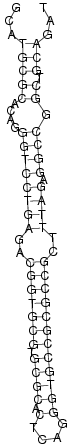

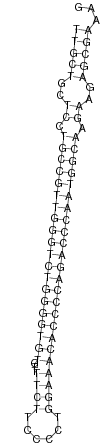

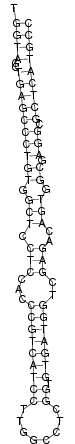

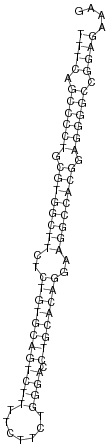

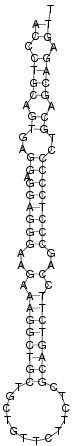

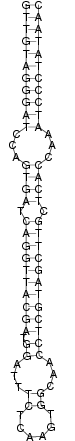


**The secondary structures above are arranged from left to right in the following order: novel_miR_10, novel_miR_12, novel_miR_14, novel_miR_17, novel_miR_18, novel_miR_20, novel_miR_21, novel_miR_22 and novel_miR_23.**


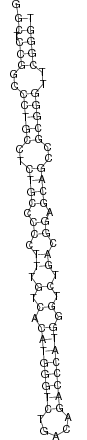

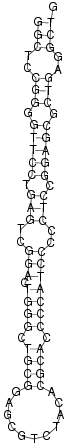

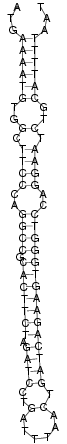

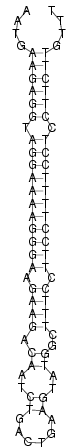

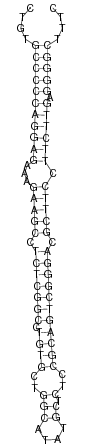

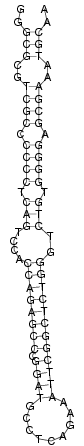

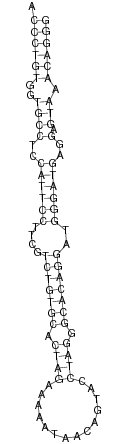

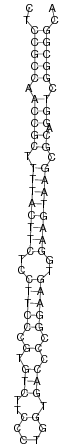

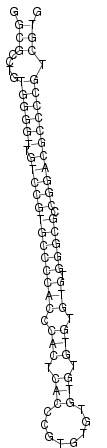


**The secondary structures above are arranged from left to right in the following order: novel_miR_24, novel_miR_25, novel_miR_26, novel_miR_27, novel_miR_28, novel_miR_29, novel_miR_30, novel_miR_31 and novel_miR_32.**


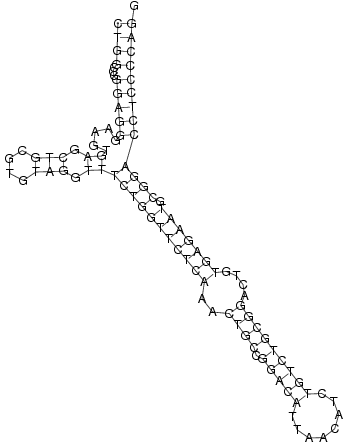

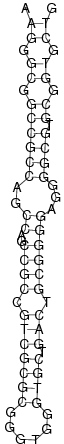

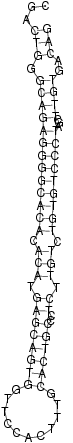

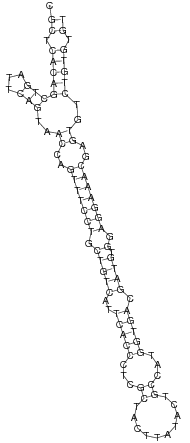

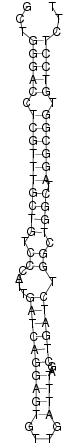

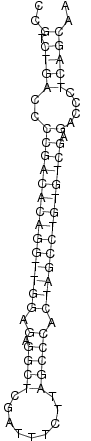


**The secondary structures above are arranged from left to right in the following order: novel_miR_34, novel_miR_35, novel_miR_36, novel_miR_37, novel_miR_38 and novel_miR_41.**


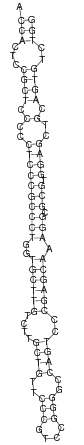

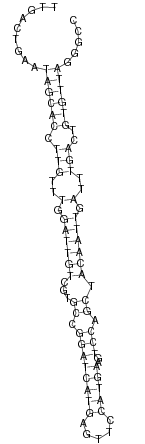

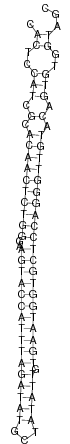

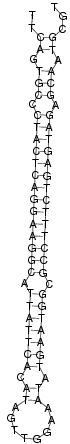

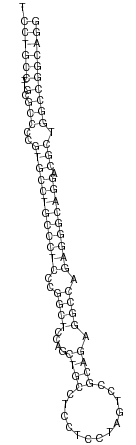

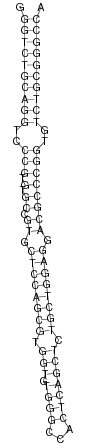

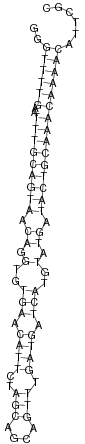

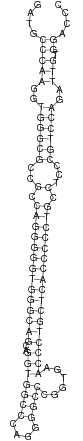

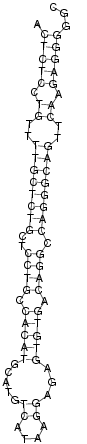


**The secondary structures above are arranged from left to right in the following order:** **novel_miR_42, novel_miR_43, novel_miR_44, novel_miR_45, novel_miR_46, novel_miR_47, novel_miR_48, novel_miR_49 and novel_miR_50.**


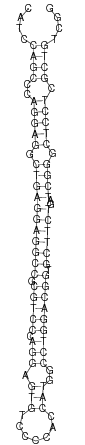

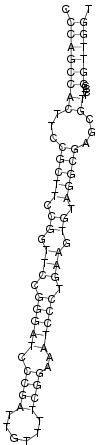

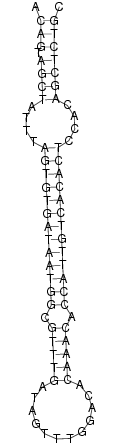

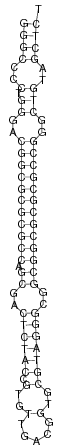

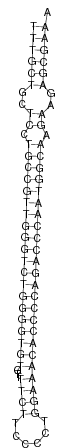

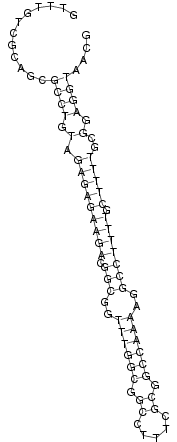

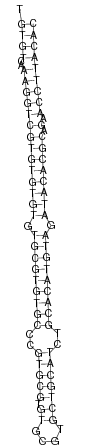

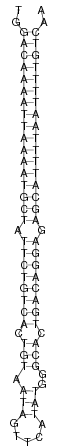

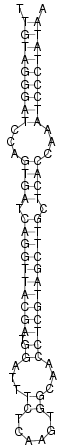


**The secondary structures above are arranged from left to right in the following order:** **novel_miR_51, novel_miR_52, novel_miR_53, novel_miR_55, novel_miR_56, novel_miR_57, novel_miR_58, novel_miR_59 and novel_miR_60.**


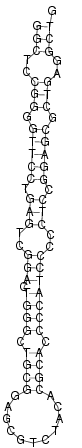

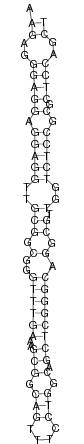

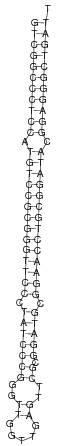

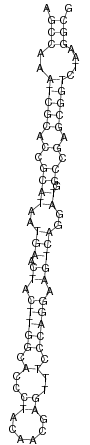

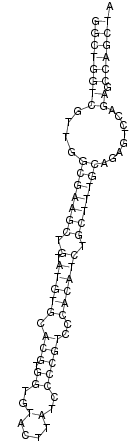

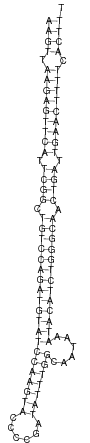

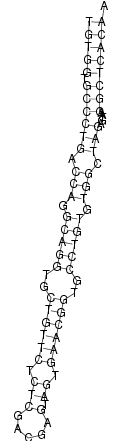

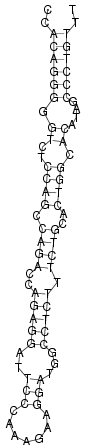

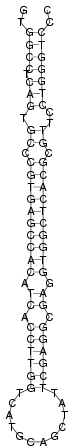


**The secondary structures above are arranged from left to right in the following order:** **novel_miR_61, novel_miR_62, novel_miR_63, novel_miR_64, novel_miR_65, novel_miR_66, novel_miR_67, novel_miR_68 and novel_miR_69.**


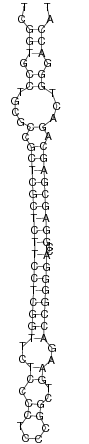

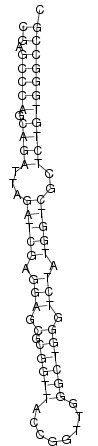

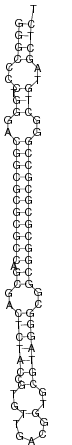

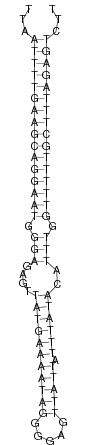

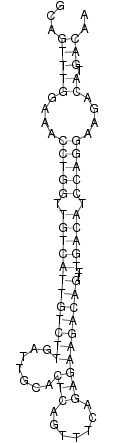

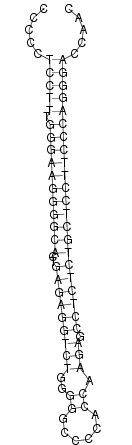

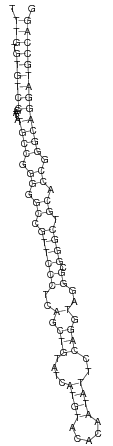

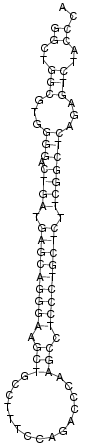


**The secondary structures above are arranged from left to right in the following order: novel_miR_70, novel_miR_71, novel_miR_72, novel_miR_73, novel_miR_74, novel_miR_75, novel_miR_76 and novel_miR_77.**


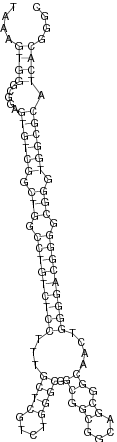

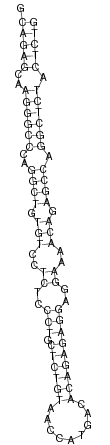

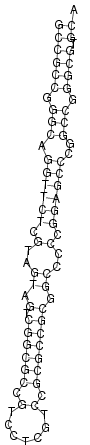

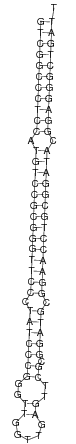

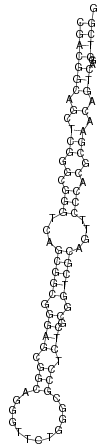

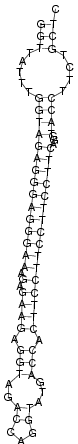

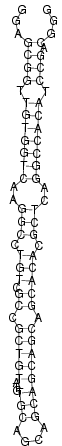

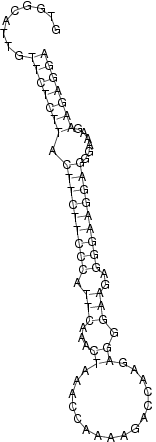


**The secondary structures above are arranged from left to right in the following order: novel_miR_78, novel_miR_79, novel_miR_80, novel_miR_81, novel_miR_82, novel_miR_83, novel_miR_84 and novel_miR_85.**


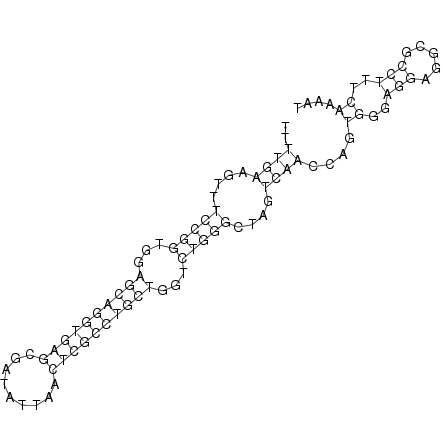

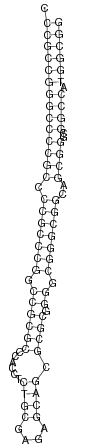

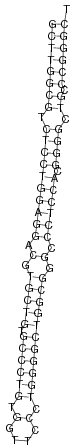

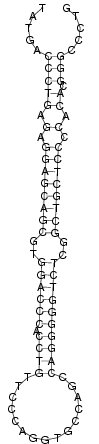


**The secondary structures above are arranged from left to right in the following order: novel_miR_86, novel_miR_87, novel_miR_89 and novel_miR_90.**


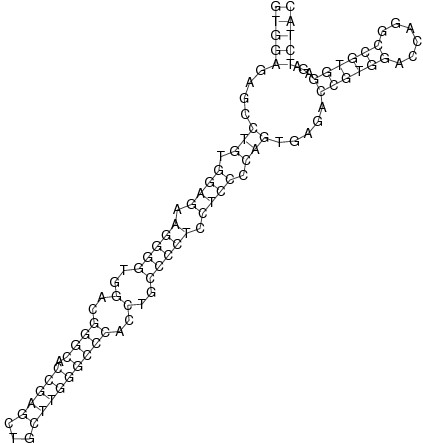

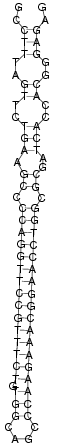

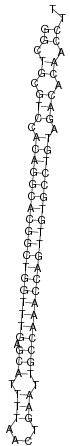

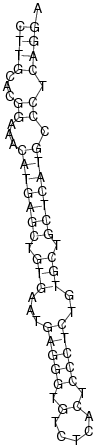


**The secondary structures above are arranged from left to right in the following order: novel_miR_91, novel_miR_92, novel_miR_93 and novel_miR_94.**


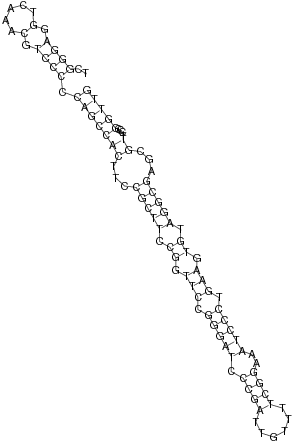

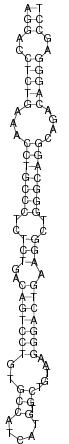

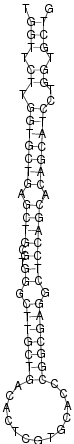

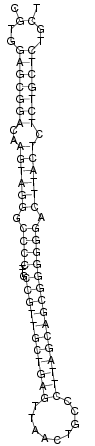

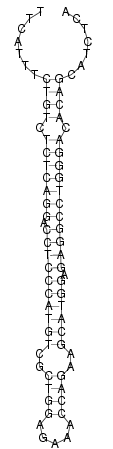

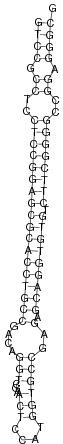

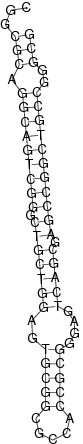


**The secondary structures above are arranged from left to right in the following order: novel_miR_95, novel_miR_96, novel_miR_98, novel_miR_99, novel_miR_100, novel_miR_101 and novel_miR_102.**


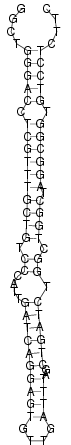

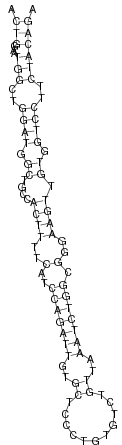

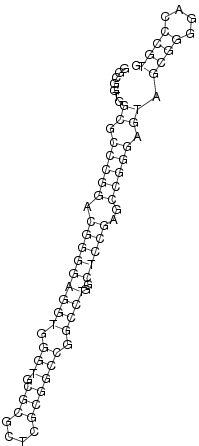

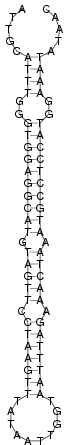

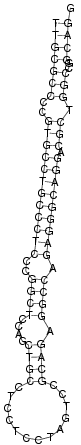


**The secondary structures above are arranged from left to right in the following order: novel_mir_103, novel_miR_104, novel_mir_106, novel_miR_107 and novel_miR_108.**
